# Chokeberry Anthocyanin Extract as Pancreatic *β*-Cell Protectors in Two Models of Induced Oxidative Stress

**DOI:** 10.1155/2015/429075

**Published:** 2015-05-31

**Authors:** Dumitriţa Rugină, Zoriţa Diaconeasa, Cristina Coman, Andrea Bunea, Carmen Socaciu, Adela Pintea

**Affiliations:** ^1^Faculty of Veterinary Medicine, University of Agricultural Sciences and Veterinary Medicine, Mănăştur 3-5, 400372 Cluj-Napoca, Romania; ^2^Faculty of Food Science and Technology, University of Agricultural Sciences and Veterinary Medicine, Mănăştur 3-5, 400372 Cluj-Napoca, Romania; ^3^Faculty of Animals Breeds and Biotechnology, University of Agricultural Sciences and Veterinary Medicine, Mănăştur 3-5, 400372 Cluj-Napoca, Romania

## Abstract

The aim of this study was to investigate the protective effects of a chokeberry anthocyanin extract (CAE) on pancreatic *β*-cells (*β*TC3) exposed to hydrogen peroxide- (H_2_O_2_-) and high glucose- (HG-) induced oxidative stress conditions. In order to quantify individual anthocyanins high performance liquid chromatography (HPLC) coupled to photodiode array (PDA) was used. The identification of the fragment ion pattern of anthocyanins was carried out by electrospray ionization mass spectrometry (LC-ESI-MS). The results showed that physiologically achievable concentrations of CAE (1, 5, and 10 *μ*M) protect *β*TC3 against H_2_O_2_- and HG-induced cytotoxicity. Antioxidant enzymes such as superoxide dismutase (SOD), catalase (CAT), and glutathione peroxidase (GPx) were increased in pancreatic *β*-cells pretreated with CAE compared to cells exposed to the prooxidant agents. GSH levels initially reduced after exposure to H_2_O_2_ and HG were restored by pretreatment with CAE. Insulin secretion in *β*TC3 cells was enhanced by CAE pretreatment. CAE restored the insulin pool and diminished the intracellular reactive oxygen species level in glucose-induced stress condition in *β*TC3 cells. These results demonstrate that anthocyanins from CAE were biologically active, showing a secretagogue potential and an antioxidative protection of enzymatic systems, conferring protection against H_2_O_2_ and glucose toxicity in *β*TC3 cells.

## 1. Introduction

In type 2 diabetes mellitus a chronic exposure of *β*TC3 cells to HG levels leads to their irreversible failure causing glucose toxicity via increased oxidative stress [1]. Oxidative stress can be defined as an imbalance between the antioxidant defense system and the production of reactive oxygen species such as superoxide anion, hydroxyl radical, and hydrogen peroxide. Disturbances of the antioxidant defense system in diabetes involve the alteration of antioxidant enzymes [2] and impaired glutathione metabolism [3]. A promising strategy is to prevent oxidative stress imbalance by natural products with antioxidant potential that may act as free radical scavengers in pancreatic *β*-cells. Some of the previously reported studies investigating the use of different plants, plant extracts, or bioactive natural compounds as potential antidiabetics were reviewed in a recent publication [4].

Anthocyanins are polyphenolic compounds which have been shown to exhibit pharmacological properties, such as anti-inflammatory, antitumor, and antioxidant activities [5, 6]. Long-term administration of berry-derived supplements, including anthocyanins, could inhibit the development of the early stages of some diabetic complications [7]. There are a number of* in vitro* studies reporting the anthocyanins protecting effect on pancreatic *β*-cells [6, 8, 9]. Anthocyanins from Chinese bayberry extract were found to protect pancreatic *β*-cells INS-1 against induced necrosis and apoptosis, via ERK1/2- and PI3K/Akt-mediated heme oxygenase-1 upregulation [6]. Jayaprakasam et al. (2005) demonstrated the ability of cyanidin-3-*O*-glucoside and delphinidin-3-*O*-glucoside to stimulate the insulin secretion from rodent pancreatic *β*-cells [8]. Using multiple cell-based bioassays, the potentially antidiabetic effects and the insulin-like activity of* Vaccinium angustifolium* were demonstrated [9]. There are some experiments on streptozotocin-induced diabetic rat model that reported the antioxidative and hypoglycemic properties of the chokeberry fruit extract [10, 11].

Anthocyanins are absorbed in blood in an intact form, so they can reach various tissues and can modulate metabolic changes in the body [12, 13]. After an anthocyanin intake, their concentrations found in plasma were very low [14]. Some pharmacokinetic data suggested that the concentrations of anthocyanins in plasma of rats were around 2 *μ*mol/L, after a diet enriched in chokeberry, bilberry, and grape, as equivalent of 3.85 g cyanidin-3-*O*-galactoside/kg, administered for 14 weeks [15]. Regarding the bioavailability of chokeberry anthocyanins, it was reported that their plasma concentrations in humans after consuming a chokeberry extract were 591 nmol/L. These concentrations were reached in about 2 h after consumption [16]. The total concentration of anthocyanins in human plasma after chokeberry juice consumption was about 32.7 nmol/L and was reached in about 1.3 h [17].

The present study was designed to prove the protecting role of a chokeberry enriched anthocyanin fraction upon pancreatic *β*-cells, using two models of oxidative-induced stress. The physiologically achievable concentrations of CAE (1, 5, and 10 *μ*M) were administered to *β*TC3 for 24 h. The CAE behavior towards the antioxidant defense system (SOD, CAT, GPx, and GSH), the insulin secretion, and the intracellular reactive oxygen species (ROS) was assessed in H_2_O_2_- and HG-induced stress conditions in *β*TC3 cells.

## 2. Materials and Methods

### 2.1. Reagents

The standard compounds, including cyanidin-3-*O*-galactoside (90% purity), cyanidin-3-*O*-glucoside (95% purity), cyanidin (95% purity), 6-hydroxy-2,5,7,8-tetramethylchroman-2-carboxylic acid (Trolox) (98% purity), 2,2-azinobis(3-ethylbenzothiazoline-6-sulfonic acid) diammonium salt (ABTS) (98% purity), 2,9-dimethyl-1,10-phenanthroline (Neocuproine) (99% purity), potassium persulfate (99% purity), acetonitrile, formic acid, ethanol, methanol, and dimethyl sulfoxide (DMSO), were obtained from Sigma-Aldrich (Darmstadt, Germany). Cyanidin-3-*O*-galactoside and cyanidin-3-*O*-arabinoside standards were bought from Polyphenols (Sandnes, Norway). HCl, NaNO_2_, H_2_O_2_, and CuCl_2_ were purchased from Merck (Darmstadt, Germany). Fetal bovine serum (FBS), Lonza, Dulbecco's Modified Eagle Medium (DMEM), and 3-(4,5-dimethylthiazol-2-yl)-2,5-diphenyltetrazolium bromide (MTT) were purchased from Lonza Group Ltd. (Basel, Switzerland). Glutamine, penicillin and streptomycin, and amphotericin were purchased from Sigma Chemical Co. (St. Louis, MO). The water used for experiments was treated in a Milli-Q water purification system.

### 2.2. Extraction of Anthocyanins and Nonanthocyanins Phenolics

The fruits of* Aronia melanocarpa* Nero cultivar were collected in the middle of August at a plantation near Cluj-Napoca (Romania) and preserved at −20°C, immediately after harvest. Anthocyanin and nonanthocyanin compounds were extracted by homogenization of chokeberries (5 g) in methanol containing HCl (0.3%) using a homogenizer (Miccra D-9 KT Digitronic, Germany) and then concentrated at 35°C under reduced pressure (Rotavapor R-124, Buchi, Switzerland). Afterwards, the extract was fractioned following the procedure described by [18]. Briefly, the aqueous extract (50 *μ*L) was filtered through a C-18 Sep-Pak cartridge (sorbent mass = 50 mg) (Waters Corp., Milford, MA), previously activated with methanol followed by 0.01% aqueous HCl. Anthocyanins and other polyphenolics were adsorbed onto the column while sugars, acids, and other water-soluble compounds were removed by washing with 2 volumes of 0.01% aqueous HCl. The second fraction, containing polyphenols (other than anthocyanins), was subsequently eluted with 2 volumes of ethyl acetate. And a third fraction, enriched in anthocyanins, was eluted with 4 volumes of methanol containing 0.01% HCl. This last fraction, named CAE, was evaporated by a rotatory evaporator under vacuum, at 35°C.

### 2.3. HPLC-ESI-MS Analysis of Anthocyanins

Analyses were performed on an Agilent Technologies 1200 HPLC system (Chelmsford, MA, USA) equipped with G1311A Quaternary Pump, G1322A degasser, G1329A autosampler, and G1315D photodiode array (PDA) detector and a Luna Phenomenex C-18 column (5 *µ*m, 25 cm × 4.6 mm) was used.* In-line* MS data were recorded by directing the LC flow to a Quadrupole 6110 mass spectrometer (Agilent Technologies, Chelmsford, MA) equipped with an ESI probe. Most settings were optimized using Flow Injection Analysis (FIA) (Agilent ChemStation) via automatic tuning with cyanidin-3-*O*-galactoside in positive ion mode. The mobile phase consisted in solvent A, formic acid (4.5%) in bidistilled water, and solvent B, acetonitrile. The gradient elution system was 10% B, 0–9 min; 12% B, 10–17 min; 25% B, 18–30 min; 10% B, 31–35 min. The flow rate was 0.8 mL/min and the analyses were performed at 35°C temperature. The anthocyanins elution order was monitored at 520 nm wavelength. For the mass spectrometry (MS) analysis, the positive ion mode scanning (ESI^+^) and fragmentation system, from* m/z* 260 to 1000, were used. The measurements were performed in the positive mode with an ion spray voltage of 3000 V, fragmentor of 70 eV and 130 eV, drying gas temperature of 350°C, gas flow (N2) of 8 L/min, and nebulizer pressure of 40 psi. The anthocyanin quantification was performed by using cyanidin-3-*O*-galactoside as pure standard and the weight correction factors. The weight correction factors were calculated as the ratio between molecular weights of the each quantified anthocyanin and the molecular weight of cyanidin 3-*O*-galactoside.

### 2.4. Antioxidant Activity

#### 2.4.1. Cupric Reducing Antioxidant Potential (CUPRAC) Assay and Scavenging Effect on ABTS Radical

The cupric ion reducing antioxidant potential of CAE was determined according to a method reported before [19]. The scavenging ability of chokeberry extracts against radical anion ABTS^•+^ was determined according to the procedure described by Arnao et al. (2001) [20]. The absorbances were recorded using a spectrophotometer (JASCO V-630 series, International Co., Ltd., Japan) against the blank reagent. The standard curve was prepared using different concentrations of Trolox and the results were expressed as *μ*mol TE/g FW.

### 2.5. Cell Culture and Proliferation

The mouse pancreatic *β*-cell line TC3 was maintained in Dulbecco's Modified Eagle Medium (DMEM) containing 5 mM glucose supplemented with 10% fetal bovine serum, 4 mM glutamine, 1% penicillin and streptomycin at 37°C, 5% CO_2_, and 95% relative humidity. The pancreatic *β*-cells were plated (1 × 10^4^ cells/well) for 24 h in 96-well microplates. CAE concentrations of 1, 5, and 10 *μ*M Cy-3-gal equivalents were used as treatment in pancreatic *β*-cells for 24 h. Stress conditions were induced by HG (50 and 100 mM) administered for 24 h or H_2_O_2_ (70 *μ*M) administered for 30 min, prior to each assay. The stock solution of CAE (395 *μ*g/mL) was prepared in deionized sterile water and further filtered through a 0.22 *μ*m Millipore filter.

The number of viable cells was determined with the 3-(4,5-dimethylthiazol-2-yl)-2,5-diphenyltetrazolium bromide (MTT) cell proliferation reagent. This method uses the property of viable cells to reduce MTT reagent into a colored formazan. Cells were washed with PBS and incubated with MTT solution (0.5 mg/mL) for 1 h. The absorbances of formazan solubilized by DMSO were read at 550 nm, respectively, at 630 nm (for background) using the microplate plate reader HT BioTek Synergy (BioTek Instruments, USA).

### 2.6. Antioxidant Enzymes Activity and Reduced Glutathione

*β*TC3 cells (5 × 10^5^ cells/well) were seeded on 6-well plates and cultured in complete medium for 24 h. The medium was then replaced with complete medium containing or not containing CAE at various concentrations (1, 5, and 10 *μ*M Cy-3-gal) in presence or absence of the prooxidant agents H_2_O_2_ and HG. After completing the treatment, cell extracts were prepared according to manufacturer's instruction and for each parameter the protein content was determined by the bicinchoninic acid assay (Sigma, St. Louis, USA). For all antioxidant enzymes, the activity was expressed on mg protein. Catalase (CAT), glutathione peroxidase (GPx), superoxide dismutase (SOD) activities, and glutathione reduced (GSH) were performed according to instruction assay kits (Cayman Chemical Company, Michigan, USA).

### 2.7. Intracellular Reactive Species Assay

Intracellular reactive oxygen species (ROS) assay is based on the oxidation of 2′,7′-dichlorodihydrofluorescein (DCHF) by intracellular peroxides, forming the 2′,7′-dichlorofluorescein (DCF). Fluorescence of this compound was measured by a BioTek microplate reader, at the excitation 485/10 nm and the emission 528/20 nm at 37°C, after 4 h [21].

### 2.8. Insulin Secretion

For the measurement of secreted insulin *β*TC3 cells (5 × 10^5^ cells/well) were seeded on a 6-well plate and treated according to the protocol presented above. Before performing the assay the culture medium was removed and replaced with Krebs-Ringer buffer (KRB; 129 mM NaCl, 4.8 mM KCl, 1.2 mM MgSO_4_, 1.2 mM KH_2_PO_4_, 2.5 mM CaCl_2_, 5 mM NaHCO_3_, 10 mM HEPES, and 0.1% BSA) containing 2.5 mM glucose and 2% FBS for 30 min at 37°C and then it was collected from each well and subjected to insulin secretion measurement according to manufacturer's instructions (Mercodia Ultrasensitive Mouse Insulin ELISA, Uppsala, Sweden).

### 2.9. Statistical Analysis

Statistical analysis was performed using the Dunnett multiple comparison test of GraphPad Prism version 5.00. Registered data represented the mean values and standard deviations (SD) of three experiments, considering three levels of significance (*∗* meant significant *P* < 0.05, *∗∗* meant very significant *P* < 0.01, and *∗∗∗* meant extremely significant *P* < 0.001).  Figure 2 was elaborated using Chemdraw Ultra 13 software.

## 3. Results and Discussion

### 3.1. Quantitative Analysis of Individual Anthocyanin Components from CAE, as Determined by HPLC-DAD-ESI-MS

**Figure 1:**
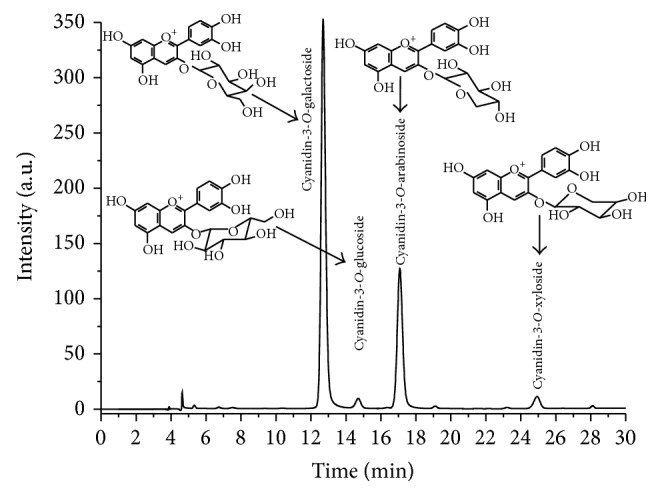
HPLC chromatogram of chokeberry anthocyanin-rich fraction (CAE). The HPLC-PDA chromatogram was registered at the wavelength of 520 nm. The chromatogram revealed that peaks correspond to the four anthocyanins, as nominated in the figure.

Anthocyanins identification and peak assignment were based on their retention times compared with pure standards, MS data, and published information. As can be seen in Figure 1, by HPLC-PDA analysis recorded at 520 nm, four peaks were identified. By HPLC-MS, each peak was identified, namely, cyanidin-3-*O*-galactoside (1) with* m/z* = 449, cyanidin-3-*O*-glucoside (2) with* m/z* = 449, cyanidin-3-*O*-arabinoside (3) with* m/z* = 419, and cyanidin-3-*O*-xyloside (4), with* m/z* = 419. The ESI-MS profiles of these compounds present specific molecular ions M^+^ and the fragment resulting from the loss of the sugar molecule 287* m/z* corresponds to the molecular ion of cyanidin aglycone. The total anthocyanin content was 1319.18 mg/100 g FW (Table 1). Cyanidin-3-*O*-galactoside was found to be the major anthocyanin present in the CAE accounting around 69% of all anthocyanins. Similar amounts of total anthocyanins were reported for chokeberries by other authors, 1478.1 mg/100 g FW [22], 1958 mg/100 g DW [23].

### 3.2. Antioxidant Activity of CAE

Two Single Electron Transfer (SET) based assays ABTS and CUPRAC were used to measure the potential of antioxidants to reduce an oxidant.

The ABTS assay uses K_2_S_2_O_8_ as oxidant and measures the antioxidants ability to scavenge the ABTS^+•^ radical. In our previous study three different* Aronia *sp. cultivars were evaluated for their* ex vivo* antioxidant activity and their potential to scavenge ABTS^+•^ and showed values between 95 and 170 *μ*mol TE/g FW [24]. However* Aronia melanocarpa* Nero cultivar used in these experiments showed a higher radical scavenging potential of 180 *μ*mol TE/g FW (Table 1). The values obtained for the scavenging activity toward ABTS^+•^ radical are similar to those reported in a recent study, that is, 37.44 mg TE/g sample [25]. Considering the water content of berries to be around 85% [26], our data are in the same concentrations range with the scavenging effect expressed, 439.4 *μ*M Trolox/100 g dry weight [23].

The CUPRAC assay is carried out at pH 7.0, close to physiological pH, and the reaction assay involves fast kinetics [27]. The ability of CAE to reduce cupric ion (Cu^2+^), as shown in Table 1, was determined to be 200 *μ*mol TE/g FW. In a previous study we obtained for other chokeberry cultivars values from 150 to 232 *μ*mol TE/g FW [24]. Recently Sariburun et al. (2010) reported CUPRAC values ranging between 47.1 and 127.9 *μ*mol TE/g FW for some raspberry and blackberry cultivars [28]. To the best of our knowledge, there is no literature data about cupric reducing antioxidant potential of similar chokeberry anthocyanin-rich extracts.

### 3.3. Pancreatic *β*-Cell Proliferation after CAE Treatment in Oxidative Stress-Induced Conditions

In order to elucidate the protective effects of CAE in the presence of the prooxidant agents, HG and H_2_O_2_, we first assessed the cell proliferation. The working H_2_O_2_ concentration was chosen from a survey curve of *β*TC3 cells treated with different H_2_O_2_ concentrations ranging from 0 to 600 *μ*M. The IC_50_ concentration of 70 *μ*M was used in this experiment as stressor agent for the *β*TC3 cells. Other previously published articles on *β*-pancreatic cells have used H_2_O_2_ in the range 200–400 *μ*M [29, 30].

With respect to the glucose concentration used, some authors have reported concentrations between 5.6 and 30 mM as oxidative stress inducer [31, 32]. In a recent study, a high dose of glucose (150 mM) was used as prooxidant agent due to a *β*-pancreatic MIN6 cell viability decreased by only 26% [33]. Our working concentrations of glucose were chosen after a preliminary assay, observing that the *β*-pancreatic TC3 cell viability after 24 h was reduced with only 20% by 50 mM and with 34% by 100 mM glucose in culture medium.

The CAE administration at physiological doses (1, 5, and 10 *μ*M Cy-3-gal) increased the *β*-pancreatic TC3 cell proliferation with about 23%, 18%, and 8%, respectively, after 24 h of treatment. As shown in Figure 3, the CAE treatment in the presence of 50 mM glucose slightly increased the cell viability. The treatment with low doses of CAE in the HG-induced oxidative stress using 100 mM glucose enhanced the cell proliferation comparing to 100 mM glucose controls without CAE (Figure 3). Meanwhile, CAE in the presence of H_2_O_2_ stress inducer stimulated the *β*-pancreatic TC3 cell proliferation. According to cell proliferation data it could be concluded that CAE had an proliferative potential on *β*-pancreatic TC3 cells in oxidative stress-induced conditions.

### 3.4. *In Vitro* Antioxidant Activity of Pancreatic *β*-Cells CAE Treated in Stress Conditions

**Figure 2:**
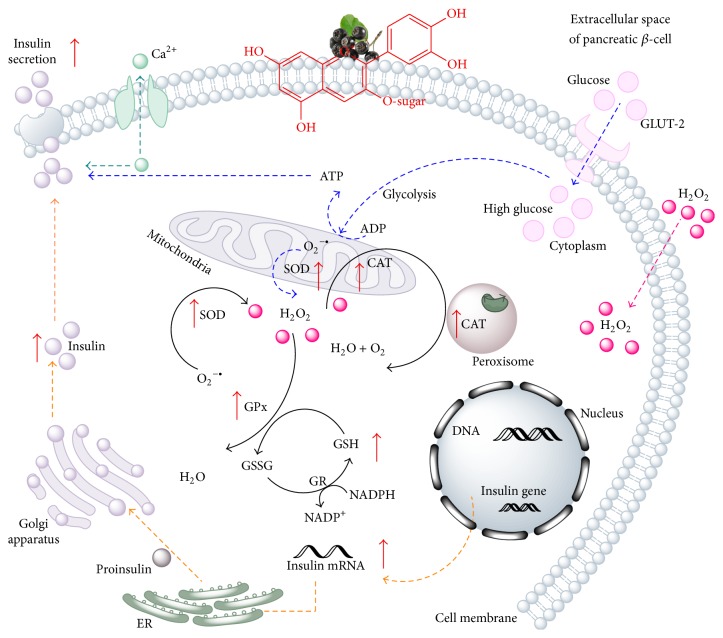
Schematic illustration of CAE-stimulated antioxidant enzyme and insulin secretion pathway in pancreatic *β*-cells in HG and H_2_O_2_ stress conditions. Glucose enters the cell across the plasma membrane through glucose transporters, like GLUT-2 for rodent *β*-cells. Once inside the cell the metabolic process of glucose called glycolysis begins. ATP is the end product of glycolysis. Further reactions take place in mitochondria where more ATP is produced. In connection with the electron transport chain the superoxide anion results. Cytosolic or mitochondrial forms of SOD catalyze the conversion of superoxide anion (O_2_^•−^) to molecular oxygen (O_2_) and hydrogen peroxide (H_2_O_2_). Unlike glucose, H_2_O_2_ administered to cells diffuses through the cell membrane. The resulting H_2_O_2_ from glucose metabolism and the extracellular H_2_O_2_ are then metabolized to harmless water and oxygen by CAT and GPx. CAE increases the activity of SOD, CAT, and GPx, so the more active the metabolism of H_2_O_2_ is, the less the reactive species are formed in the cell. GSH levels are reduced by H_2_O_2_ and HG and restored by CAE. It is possible that the anthocyanins increase the insulin gene expression, because the CAE treatment attempts to restore the pool of insulin. Before being secreted out of the cell insulin is synthesized and converted to proinsulin in the endoplasmic reticulum (ER) folded and transported to the Golgi apparatus. Anthocyanins from CAE could also influence the opening of the voltage-gated Ca^2+^ channels, leading to an increased fusion of insulin granule with the cell membrane.

**Figure 3:**
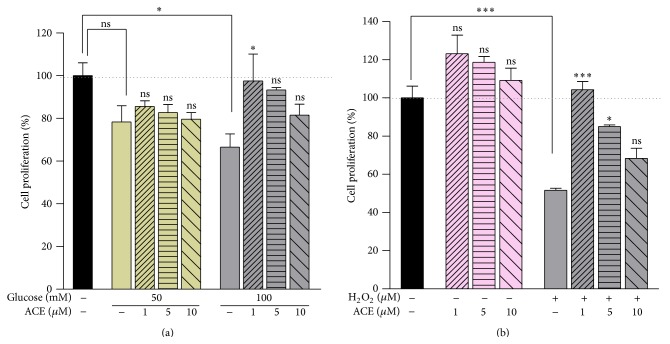
Cell viability assessed by MTT assay. CAE increases the proliferation of *β*TC3 cells treated in (a) HG-induced stress for 24 h and (b) H_2_O_2_-induced stress for 15 min. Data are expressed as mean ± SEM (*n* = 5). Statistically significant differences: ^*∗*^ *P* < 0.05, ^*∗∗*^ *P* < 0.01, and ^*∗∗∗*^ *P* < 0.001 compared with control.

The enzymatic antioxidants such as superoxide dismutase (SOD), catalase (CAT), and glutathione peroxidase (GPx) and the nonenzymatic antioxidant (GSH) can act together to attenuate the ROS produced by HG and H_2_O_2_ administration to *β*-pancreatic cells. Cytosolic or mitochondrial forms of SOD catalyze the conversion of superoxide anion (O_2_^•−^) to molecular oxygen (O_2_) and hydrogen peroxide (H_2_O_2_). This resulting H_2_O_2_ is then metabolized to harmless water and oxygen by CAT and GPx. SOD and CAT do not require any cofactors to function (Figure 2). Unlike SOD and CAT, GPx needs some cofactors (reduced glutathione, NADPH, and glucose 6-phosphate) and proteins (glutathione reductase and glucose-6-phosphate dehydrogenase) in order to operate effectively [34]. In consequence, ROS does not have a direct influence on GPx, but its function could be abolished due to a disorder in the glutathione system.

**Figure 4:**
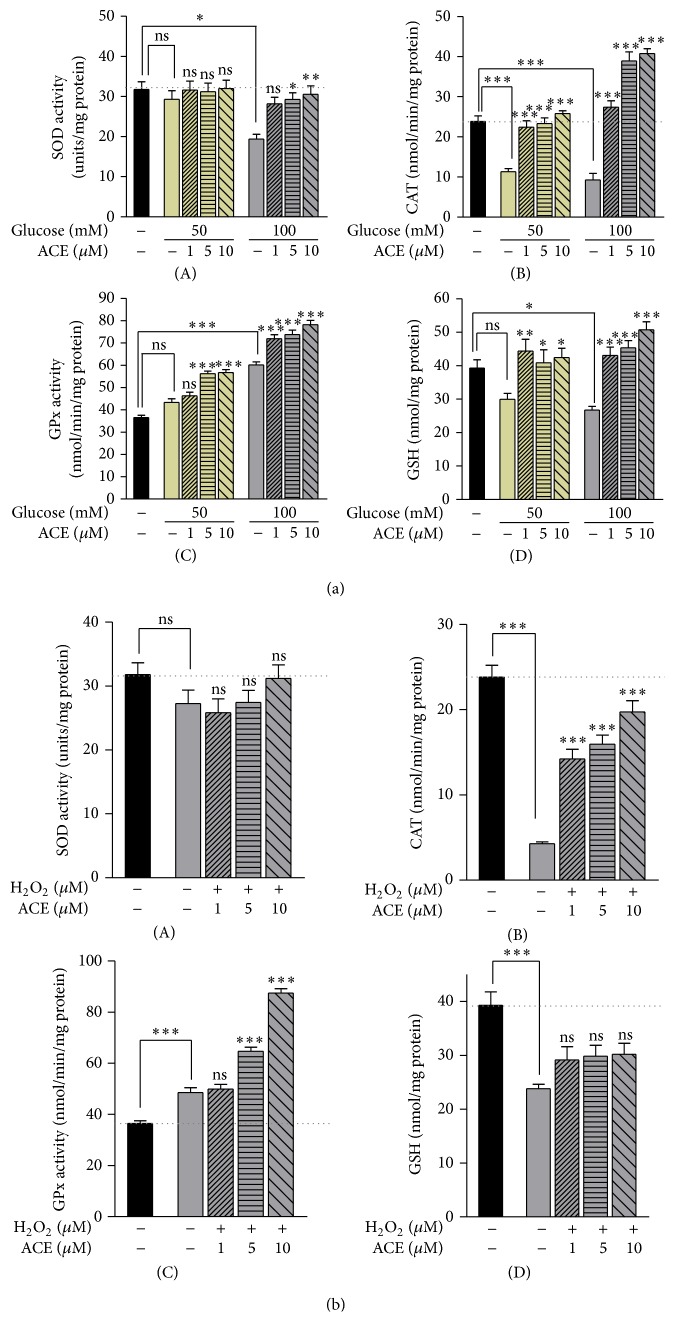
Effect of CAE on the levels of antioxidant enzyme activities in *β*TC3 cells in stress-induced conditions. (a) HG-induced stress for 24 h: (A) SOD; (B) CAT; (C) GPx; (D) GSH and (b) H_2_O_2_-induced stress for 15 min: (A) SOD; (B) CAT; (C) GPx; (D) GSH. Data are expressed as mean ± SEM of two independent determinations. Statistical analyses: ^*∗*^ *P* < 0.05 versus the control.

In the presence of H_2_O_2_ and HG as stress agents, the CAE induced a strong increase of CAT, GPx activities, and GSH content in *β*-pancreatic TC3 cells, as shown in Figures 4(a) and 4(b). Also, a slight increase in the activity of SOD was observed in CAE-treated *β*-pancreatic cells compared to untreated cells; see Figures 4(a)(A) and 4(b)(A). Since CAT and GPx have different enzymatic characteristic, both of them are H_2_O_2_-inactivating enzymes. GPx inactivates the H_2_O_2_ substrate with high affinity in a continuous rate of oxygen free radical production, whereas CAT has a lower affinity in a higher continuous rate of free ROS production [35]. A possible explanation for the statistically significant increased activities of CAT and GPx by CAE treatment in oxidative stress-induced conditions (50 mM HG and 70 *μ*M H_2_O_2_ in *β*-pancreatic TC3 cells) could be the high rate production of their substrate H_2_O_2_ and a scarce existence of superoxide anion (O_2_^•−^) (Figures 4(a)(B) and 4(a)(C)).

Hence, *β*-pancreatic cells could be an easy target for ROS, possibly because of their low expression of SOD, catalase, and GPx genes (as reviewed by Robertson et al. (2003)) [36]. Compared to the liver enzymatic system, pancreatic islets contain 1% catalase, 2% GPx, and 29% SOD1 activities (reviewed in Ježek et al. work (2012)) [37]. However, our results show that the CAE is acting when a scavenging excess of free radicals was produced under induced stress conditions to ensure the *β*-pancreatic cells protection. Even though the enzymatic antioxidant defense system is considered to be poorly expressed in *β*-pancreatic cells, it can contribute to the “housekeeping” processes.

The efficiency of the chokeberry rich anthocyanin fraction towards SOD activity was demonstrated previously; the treatment induced an increased activity into rat blood unlike the control model. In the same study the GPx activity in the rat's blood was not affected after chokeberry extract administration [11]. The effect of other anthocyanins like pelargonidin restored the levels of SOD and CAT in diabetic rats after 2 weeks of treatment [38].

### 3.5. Insulin Secretion Activity of CAE in Pancreatic *β*-Cells Exposed to Stress Conditions

It has already been proved that the insulin secretion could be diminished by oxidative stress conditions [39, 40]. Therefore, we investigated also the CAE insulin secretagogue effect in pancreatic *β*-cells exposed to HG and H_2_O_2_ stress conditions.

**Figure 5:**
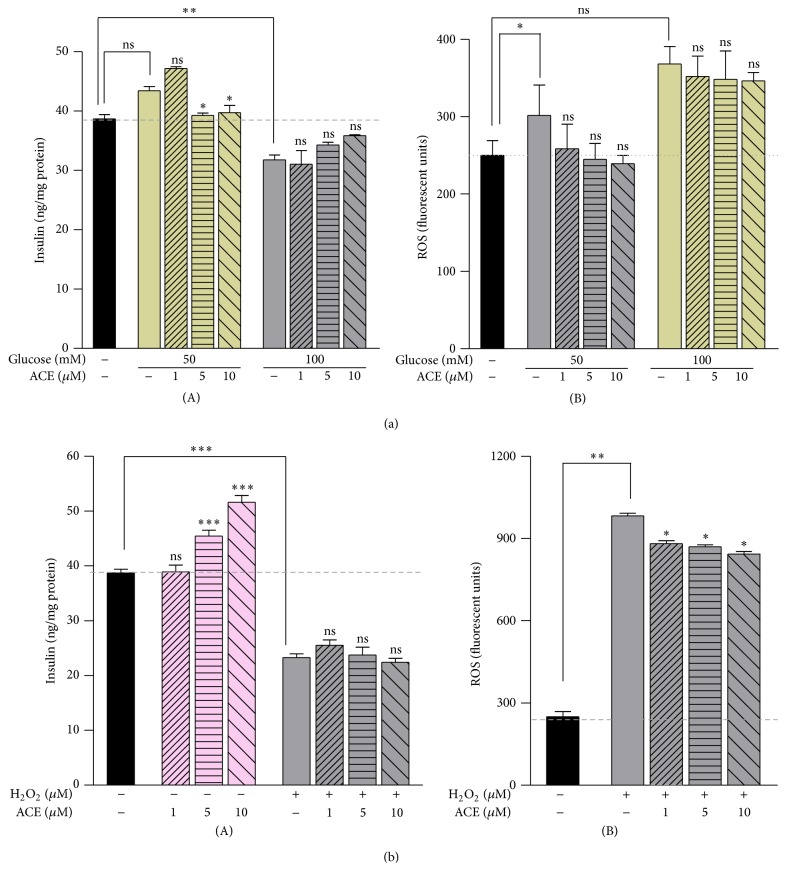
Effect of CAE on insulin secretion and intracellular reactive oxygen species (ROS) level in *β*TC3 cells in stress-induced conditions. (a) HG-induced stress for 24 h and (b) H_2_O_2_-induced stress for 15 min. Data is expressed as mean ± SEM of two independent determinations. Statistical analyses: ^*∗*^ *P* < 0.05 versus the control.

In our study the administration of CAE concentrations of 1, 5, and 10 *μ*M increased insulin secretion by 1, 9, and 18 ng of insulin/mg of protein, respectively, under basal glucose condition (Figure 5(b)(A)). The low dose of CAE (1 *μ*M) increased the insulin secretion by 4 ng of insulin/mg of protein in HG (50 mM) stress condition (Figure 5(a)(A)). In a dose-dependent manner the CAE administration attempted to restore the pool of insulin in the HG (100 mM) environment (Figure 5(a)(A)). The CAE treatment in the presence of H_2_O_2_-induced stress did not have any influence on the secreted insulin in pancreatic *β*-cells (Figure 5(b)(A)). This may be possible if the insulin gene transcription was decreased after the exposure to H_2_O_2_ of pancreatic *β*-cells [41]. The insulin secretagogue effect of anthocyanins and anthocyanidins was demonstrated on pancreatic *β*-cells INS-1 832/13 previously [8]. In the same study an increment by the cyanidin-3-*O*-glucoside treatment of the insulin secretion was recorded, from 33 ng insulin/mg protein to 46 ng insulin/mg protein in basal glucose (4 mM) conditions. Glucose-induced stress (10 mM) increased for about 4 times the insulin secretion. It was observed that higher than 10 *μ*g/mL (20 *μ*M) cyanidin-3-*O*-glucoside concentrations did not significantly improve the insulin secretion in rodent pancreatic *β*-cells INS-1 832/13 [8]. Some* in vivo* studies reported that cyanidin-3-*O*-glucoside and anthocyanin-rich extracts ameliorated the hyperglycemia and the insulin sensitivity in diabetic mice [42, 43]. Insulin gene expression was decreased by exposure to oxidative stress in HIT-T15 cells or isolated rat islets [41].

### 3.6. Intracellular Reactive Species Level after CAE Administration in Pancreatic *β*-Cells in Oxidative Stress Conditions

It is shown that HG- and H_2_O_2_-induced stress conditions cause elevated ROS levels in pancreatic *β*-cells [36, 44]. Therefore, we have examined the role of ROS in H_2_O_2_- and HG-induced stress conditions (Figures 5(a)(B) and 5(b)(B)). Cells were treated as described above and stained with the redox-sensitive fluorescent probe DCFDA, which was oxidized by intracellular ROS to the highly fluorescent DCF compound detected by the microplate reader. Figures 5(a)(B) and 5(b)(B) show an increase in the production of ROS by 100 units in 100 mM HG, respectively, and by 50 units in 50 mM glucose-induced stress, compared to the control treatment. CAE administration attempts to decrease the intracellular ROS level in stress conditions in a dose-dependent manner.

In this study we used hydrogen peroxide- and high glucose-induced toxicity in pancreatic *β*-cells as models to evaluate the antioxidant defense system behavior after administration of three physiologically achievable concentrations of chokeberry anthocyanin extract. Furthermore CAE proved to have a role in protecting pancreatic *β*-cells increasing the SOD, CAT, GPx, and GSH levels, a secretagogue potential restoring the pool of the insulin, and an antioxidant activity limiting the reactive prooxidants generation in induced stress conditions. In conclusion one can assume a specific role of CAE as a defender of pancreatic *β*-cells against the aggressive agents-induced stress conditions, providing evidence for the support of the anthocyanins usage as a preventive treatment for diabetes.

## Figures and Tables

**Table 1:** The CAE anthocyanin content (mg/100 g FW) and the antioxidant potential (*μ*mol TE/g FW).

Anthocyanins	Anthocyanin content		ABTS	CUPRAC
	mg/100 g	%	*μ*mol TE/g FW	
Cy-3-*O*-galactoside	906.9 ± 20	69.00	180.50 ± 8.10	203.80 ± 8.70
Cy-3-*O*-glucoside	14.9 ± 30	1.10		
Cy-3-*O*-arabinoside	352.4 ± 15	27.00		
Cy-3-*O*-xyloside	44.8 ± 80	3.40		
Total	**1319.18**	**100.00**		

Cy: cyanidin.
